# The violence of narrative: embodying responsibility for poverty‐related stress

**DOI:** 10.1111/1467-9566.13084

**Published:** 2020-04-06

**Authors:** Felicity Thomas, Katrina Wyatt, Lorraine Hansford

**Affiliations:** ^1^ University of Exeter Medical School Exeter UK

**Keywords:** Bourdieu, health inequalities, mental health, medicalisation, narratives, poverty

## Abstract

Narratives of self‐responsibility are pervasive in neoliberally oriented contexts, and have been found to engender feelings of shame and failure amongst those affected by poverty. Here, we use findings from research in two low‐income communities in south‐west England to examine how these narratives become embodied within people's daily lives when they intersect with systems of welfare support and the current political drive to upscale treatment for common mental health conditions. Drawing on Bourdieu's notion of symbolic violence, we examine how narratives of self‐responsibility and associated welfare reform strategies impact on the mental health of people living in economic hardship. The data show how such narratives inflict, sustain and exacerbate mental distress and suffering, and how they become naturalised and normalised by individuals themselves. We demonstrate how this situation pushes people to seek support from General Practitioners, and how clinical interactions can normalise, and in turn, medicalise, poverty‐related distress. Whilst some people actively resist dominant narratives around self‐responsibility, we argue that this is insufficient under broader sociocultural and political circumstances, to free themselves from the harms perpetuated by symbolic violence.

## Introduction

Poor, ‘working class’ neighbourhoods and the people within them have long been the subject of public scrutiny, judgement and media scorn. The narratives that are used to demean the poor are formidable, and have been deployed across recent decades by a series of governments in the UK from across the political spectrum; from notions of an ‘underclass’ during the Thatcher era, to the language of ‘exclusion’ during the Blair era, and more recently under the coalition and current Conservative government, to adages relating to a ‘broken’ and ‘lost’ country that requires punitive changes to welfare to restore economic order (McKenzie 2015).

Underpinning all such rhetoric has been what Lawler (2005) describes as a ‘narrative of lack’, in which (usually white) working class people are seen by others (middle classes, politicians, media) not only to lack material resources, but to be deficient in ‘taste’, knowledge, and what Bourdieu (2010) refers to as the ‘right’ ways of ‘being and doing’. Such thinking is clearly evident in a range of characters that have emerged in UK popular culture that typify the prejudices commonly expressed against poor or working class people. Little Britain's Vicky Pollard and Shameless's Frank Gallagher, for example, incite ‘disgust’ (Lawler 2005) by propping up stereotypes of dysfunctionality and fecklessness (Jones 2012, Valentine and Harris 2014), a message also clearly conveyed in the proliferation of reality programmes such as Channel 4's Benefits Street and Channel 5's On Benefits and Proud (Jenson 2014).

Opportunities for vitriol and stereotype have been amplified under austerity, where much of the focus of this assumed ‘lack’ or deficit has centred around notions of self‐responsibility. This can be seen reflected in neoliberal‐orientations and goals that increasingly restrict entitlement to welfare support to address what George Osborne as Chancellor described as an entrenched ‘something for nothing culture’ (Blackburn 2013) in which people in receipt of benefits are seen to ‘shamelessly’ expect to be provided for whilst others to go out to work. In a context in which success is largely measured through personal economic achievement, the narratives propagated by politicians and the media act to ‘validate each other and control the circulation of competing discourses’ (Bourdieu 2018: 627), in turn fuelling a situation in which those experiencing poverty and disadvantage become cast as moral failures, denied recognition (Renault 2017) and seen as devoid of value and worth (Charlesworth 2000, Skeggs 2004).

As Walker (2014) has argued, rendering ‘welfare’ as a term of abuse has also facilitated a range of reforms that have led to wide‐scale reductions in entitlements across the UK in recent years. Amongst other things, this includes a cap on the benefits available to an individual or household, the introduction of a controversial ‘simplified’ benefit payment system through the roll‐out of ‘Universal Credit’, and the imposition of the ‘bedroom tax’, whereby people living in social housing that is deemed to surpass their basic needs now have to pay for any ‘spare’ bedrooms within the property.

Whilst the current narratives in use against poor and working class people are not in themselves unique, they are being played out alongside broader changes in the conceptualisation and treatment of common mental health issues (depression and anxiety). The provision of effective treatment and support for mental health is a stated aim of the British Government (HM Government 2011), and can be seen reflected in the sharp upsurge in antidepressant prescribing and referrals to talking therapies in the past decade (Clark 2011, NHS Digital 2017). In England, the number of antidepressant items prescribed more than doubled from 33.7 million in 2006 to 64.7 million in 2016 (NHS Digital 2017). Recent analyses demonstrate particularly high levels of prescribing and use of psychoactive drugs in low‐income communities (EXASOL 2017).

One reading of this trend is that government‐provided mental health services have successfully challenged the inverse care law by ensuring that widely recommended treatments are available to all who need them. However, there is growing concern that these changes are part of an increasing trend towards the pathologisation of the everyday stresses of life lived in hardship (Mills 2015, Thomas *et al*. 2018). Framing this kind of mental distress as a mental health ‘condition’ that lies within the individual concerned has a series of important and potentially detrimental repercussions: firstly, it implies that distress caused by everyday challenges of social and economic disadvantage can be ‘corrected’ through medical or therapeutic intervention (Busfield 2011); secondly, it masks the factors that often underlie the root causes of suffering, for example, poor living conditions, unemployment, social isolation; and thirdly, it reiterates stereotypical assumptions and negative narratives around the behaviour of people living in economically disadvantaged circumstances, whilst absolving those with power from taking responsibility for the injustices caused by ongoing economic, social and health inequalities (Friedli and Stearn 2015).

Drawing on Bourdieu's notion of symbolic violence, we examine the ways that narratives of self‐responsibility and associated welfare reform strategies impact on the mental health of people living in economic hardship. We demonstrate how these narratives inflict, sustain and exacerbate mental distress and suffering, yet can also become naturalised and normalised by both community members and the health professionals that seek to support them, in turn fuelling the medicalisation of poverty‐related distress. Whilst we show how some people actively repurpose or resist dominant narratives as a protective mechanism, and as a means of creating a self‐affirming identity, we argue that such thoughts and actions are insufficient, under broader sociocultural and political circumstances, to free them from the harms perpetuated by symbolic violence.

## Symbolic violence and mental distress

Much work on contemporary systems of social classification, domination and value draws on Bourdieu's ideas around cultural capital and ‘taste’ and his associated conceptualisation of symbolic violence, with his later, more politically charged work (e.g. Bourdieu 2000, 2018), providing a particularly useful basis for understanding how social inequalities are exerted upon ‘ordinary citizens’.

Focusing on the notion of capital as a factor determining people's social position, Bourdieu's work extends Marxist thinking beyond the economic and into the more symbolic realm of cultural capital. Power therefore, is seen as deriving not only from the possession of material resources, but also from cultural and social resources. Furthermore, through the concept of symbolic capital, Bourdieu recognises that various forms of capital acquire value, prestige and legitimacy which ‘enable forms of domination which imply dependence on those who can be dominated by it, since it only exists through the esteem, recognition, belief, credit, confidence of others, and can only be perpetuated so long as it succeeds in obtaining belief in its existence’ (Bourdieu 2000: 166). In Distinction (1984), Bourdieu sets out how family and educational influences are key to the acquisition of cultural capital (including skills, taste, mannerisms and posture, material belongings and qualifications). Integral within his theory of ‘habitus’, Bourdieu (2010) argues that predispositions to certain kinds of cultural capital such as food, music and art are taught and instilled at a young age and that these tastes in turn guide people to their ‘appropriate’ social positions and practices. Those, he argues, with a high volume of cultural capital, are most likely to be able to determine what constitutes taste (and associated notions of acceptability) within society, whilst those with lower cultural capital accept this as legitimate and natural, whilst lacking the necessary means (such as the correct education or terminology) to achieve this capital themselves. Cultural capital thus plays a core role in the hierarchical structuring of class and identity, by separating those who have desirable capital from those who do not. This, in turn, acts as a major source of social inequality and suffering that is produced and maintained less by physical force than by forms of symbolic domination – the results of which he refers to as symbolic violence.

Symbolic violence constitutes the sociocultural mechanisms and relations of unequal power that exist within interpersonal interactions and relationships, and that manifest in everyday life through language, symbolism and the actions of ‘normal’ routines, enabling a sociology of power that explores both the ‘micro‐politics of everyday life and the macro‐politics of institutional exclusion’ (Topper 2001: 31). Whilst symbolic violence relates to the imposition of meaning, it also considers *whose* values and mechanisms are legitimated as producing knowledge. Bourdieu (1977) claims that power operates through the subjective ‘misrecognition’ of meanings associated with a particular action, practice or ritual, in turn, endowing it with ‘legitimacy it would not otherwise have’ in the eye of the beholder (Bourdieu 1992: 23). Symbolic violence thus begins when the ‘misrecognition [meconnaisance] implied by this recognition [reconnaissance] leads those who are dominated to apply the dominant criteria of evaluation to their own practices’ (Bourdieu and Boltanski, in Thompson 1984: 46). The naturalisation of dominant taste and its ‘misrecognition’ as normal and necessary in turn is seen to deny the working classes the means of defining their own world.

Importantly for Bourdieu, symbolic domination is hard to resist because ‘it is something you absorb like air’ (Bourdieu and Eagleton 1992), meaning that even those who benefit least – or have most to lose from the dominant power hierarchy – participate to some extent, in reproducing their own subjection (Bourdieu 2000, Bourdieu and Wacquant 1992). This is in part because the voices of the dominated are so hard to hear; but is exacerbated because when the subjugated speak to the dominant group, they ‘tend to use a borrowed discourse, the very one the dominant offer about them’ (Champagne 2018: 51). For Bourdieu (2000: 171), this form of ‘consent’ and ‘complicity’ in their own subjugation is the ‘effect of a power which is durably inscribed in the bodies of the dominated, in such a way that it is experienced as legitimate’ and ‘natural’. The power of symbolic violence therefore ‘rests precisely in its lack of visibility’ in that it is ‘unrecognisable for what it is’ and in so being, can uphold and legitimate the existing social order (Morgan and Björkert 2006: 448). At the same time, an awareness of the injustices to which the dominated are subjugated can make the violence and emotional pain experienced even more intense and distressing (Charlesworth 2001). Furthermore, when working class people do attempt to emulate middle class taste or behaviour, they can also be condemned for this, or ridiculed as brazen, their role being simply to act as a ‘foil, a negative reference point, in relation to which all aesthetics define themselves, by successive negations’ (Bourdieu 2010: 50).

In *The Weight of the World*, Bourdieu (2018) present a series of case studies to demonstrate the various ways that social and economic injustices sit alongside the ‘muted violences’ of everyday life for France's dispossessed. In a similar vein, Charlesworth's (2000, 2001) poignant ethnographic writings on working class experience in Rotherham demonstrate how enforced acceptance of the realities of an inegalitarian social order can leave people feeling ‘insignificant’, ‘debilitated’ and ‘unable to imagine any possibility of being other than they are’. This, he says, ‘sets parameters to their ways of dealing with the world … it haunts everything that working people think and say and every choice they make’ (2001: 52). In such circumstances he argues, people are without hope and belief that they can affect their future leaving people ‘condemned to a life of meaninglessness’, with time that they cannot use productively and a future that seems as a ‘vertical face they cannot begin to conquer’ (ibid: 59).

As others have described, many of the biological processes that lead to ill health are stimulated by what people think and feel about their material and social circumstances, and are particularly injurious when people feel belittled or degraded (Wilkinson 2001, Wilkinson and Pickett 2018). Thus, when people perceive themselves and the value of their worth so negatively, it influences the likelihood of them excluding themselves from public life (Bourdieu and Wacquant 1992, Putnam 2001), and also leads to extreme nervous tension and the triggering of biological stress responses (Charlesworth *et al*. 2004). This raises important questions over the ways that people conceive of the mental distress they experience, and, at a time when mental health treatment is so high on the political agenda, how people seek to respond to this distress.

This paper examines the symbolic violence intrinsic within people's everyday encounters with narratives of self‐responsibility. We demonstrate how these narratives inflict mental distress and suffering, yet can also become naturalised and normalised by individuals themselves. We show how this situation pushes people to seek support from General Practitioners (GPs), and how clinical interactions themselves can normalise, and in turn, medicalise, what are inherently social and structural issues of poverty.

## Methodology

Findings draw on DeStress, a 2&frac12;‐year research project with two low‐income urban communities (one post‐industrial, one coastal with seasonal employment) in the UK's south‐west region. Both sites represented the least affluent quintile as determined by the Indices of Multiple Deprivation (Ministry of Housing, Communities and Local Government 2015). Ethics permission was obtained from the NHS Cambridgeshire and Hertfordshire Research Ethics Committee.

The study aimed to gain insight into the ways that narratives of self‐responsibility were taken up and embodied – or alternatively, resisted – within economically disadvantaged communities; the ways these narratives and associated welfare reforms impacted on mental distress; and the way these narratives interconnected with the medicalisation and pathologisation of poverty‐related distress. The study involved 16 focus groups with 97 participants (aged 18–65) from economically disadvantaged communities to establish the source and impact of narratives of self‐responsibility within people's everyday lives (36 men, 61 women). Fifty‐seven low‐income residents (aged 18–65) who had experienced poverty‐related mental distress were also interviewed (26 men, 31 women) to understand the cause(s) of their distress, and their responses to this. Of these participants, 46 had been prescribed antidepressants (often alongside talking therapy), whilst a further four had refused the prescription offered. The remaining seven had been advised to self‐refer to talking therapy, or had chosen to avoid interaction with health services.

Potential participants were alerted to the study by community and health practitioners, social media and word‐of‐mouth and recruited through community groups and GP surgeries. Participants receiving mental health treatment at the time of the study, and participants who wanted more time to discuss their experiences were interviewed on two occasions (total interviews *n* = 80), enabling us to track responses over time and facilitating the triangulation of data. In almost all cases, study participants had lived in an economically disadvantaged area throughout their lives, though older participants in one area had also lived there at a time when it was more prosperous, and employment (albeit still low waged) was felt to have been easier to obtain. All lived on low incomes that were obtained through employment, through welfare payments, or a combination of the two. Whilst not specifically asked to define their status in terms of class, people commonly defined themselves through characteristics or inferences usually associated with being ‘working class’, including for example, the type of housing in which they lived (social housing or insecure private rental), the kinds of employment they held or sought (usually poorly paid, ‘blue collar’ or low level service‐industry work), or the kinds of cultural capital they held (e.g. qualifications) or embodied (e.g. accent and ways of speaking).

Because primary healthcare providers play a key role in diagnosing illness and act as gatekeepers to treatment (and, by proxy, to welfare), it was important to understand how they perceived and responded to patients presenting with poverty‐related mental distress. Interviews with 10 GPs (7 women, 3 men) working in the study sites and in neighbouring low‐income areas were therefore undertaken to understand the challenges they faced supporting mental health amongst patients experiencing poverty, their attitudes towards diagnosis of mental distress, and their perceptions of current treatment options.

Informal discussions were also undertaken with key service providers from health, education and social sectors to gain insight into their perceptions and experiences of working with people living with the stresses of poverty.

Audio recordings from the focus groups and interviews were professionally transcribed. All transcripts were read iteratively in light of existing social science and public health literature relating to poverty, welfare reforms and mental health. Holistic categorical context analysis focusing on thematic similarities and differences between the narratives generated within and across the data (Lieblich *et al*. 1998) and structural analysis identifying diverse narrative ‘types’ (Reissmann 1993) provided insight into the ways that narratives around poverty and mental health were conditioned and shaped by their circumstances (Holstein and Gubrium 2012). A theme relating to narratives of responsibility was identified across focus group discussions and within interviews with GPs and patients. Subthemes emerged within this category that related to the source of the narratives (e.g. media portrayal, health professionals), and how these narratives impacted on, or were perceived to impact on, people living in poverty, for example, feeling judged as a parent; pressures to seek medical help. Coding was undertaken (using NVivo 11) and cross‐checked by three team members to ensure consistency in data interpretation and to reconcile any discrepant codes.

Field data were triangulated across sources of primary data, as well as through feedback and validation with individual study participants taking part in interviews and focus groups. The research team also met regularly to synthesise the various data strands in sense‐making sessions. Additionally, data analysis and emerging findings were extensively discussed during meetings with the project Advisory Board. Comprising residents (*n* = 8) from the study sites and representatives from the health, policy and civil society sectors (*n* = 7), Advisory Board meetings proved an important mechanism for discussing the data through the diverse analytical lens (e.g. researcher, clinician, community partner) through which interpretations were being made. This not only enabled opportunities to question and interrogate the data, but also enabled us to identify emerging themes and avenues of enquiry and to validate our research findings.

## Findings

### Everyday forms of violence

Our research elicited an abundance of evidence to demonstrate how narratives reinforcing a failure of individual responsibility were played out and embodied within people's everyday lives. In discussions with representatives from key support agencies and statutory organisations, for example, it was not uncommon to hear comments that confirmed dominant rhetoric in which people living in poverty were seen as witting players in their own plight; through assertions that they were ‘not bothering to help themselves’; that their situation of precarity was directly linked to their ‘chaotic’ and ‘irresponsible’ lifestyle; or, as one professional put it, that ‘they’ [people experiencing economic hardship] ‘obviously got something out of being depressed’. Such comments, in turn, fed into a broader and persistent narrative of blame in which people were continually exposed to their perceived inadequacies and their alleged failure to take responsibility for themselves and their family. For Shirley for example, a problem with mould in her flat had been put down to her cooking the ‘wrong’ type of food, and placing her furniture against the walls in an attempt to maximise space. Similarly, for Hannah, problems of overcrowding in a small flat (condemned as unsafe by her health worker) were scorned by the housing provider, after Hannah turned down alternative accommodation which would have required her to get up steps to the entrance which she could not manage with twin babies. Common across all participant experiences was the unceasing reminder from service providers and statutory organisations that they were ‘lucky’ to be ‘given’ anything, an assertion that served to continually reinforce dominant power hierarchies and to remind people that they, and their lives, were under various forms of surveillance and commentary.

As has been reported elsewhere (e.g. Walker 2014) receiving state welfare or other forms of more localised support such as food banks, carried with it a hefty burden of shame and stigma, particularly when people were constantly made to feel that they were undeserving of this support:

If you mention you've been to a food bank or you've had to get – we've got the debt recovery order so I can't get any credit, you know, things like that – people automatically think, well that's because you spent all your money on fags and booze. (Anita)

Professional staff […] they tell you, ‘no, you don't have that [health condition], you are over‐reacting’, or ‘you are lazy’. So that makes it a little bit worse. It just digs that hole of low mood a little bit deeper so it's harder to get out of. (Carla)

Research participants explained that this was particularly apparent when dealing with Job Centre advisors, where, unless you were ‘lucky’ to have an understanding key worker, the norm was to feel ‘disbelieved’, ‘seen as scum’, and blamed for the smallest misdemeanours or, more commonly, for systemic failures and delays that clearly fell outside their control. Here, the power differential between parties was widely considered to have unpredictable and usually negative implications for those seeking support:

It's like walking on eggshells with your JSA [Jobseeker's Allowance] advisor – because if you miss anything, they do have the power to, like, ultimately mess everything up for you in one session. (Ethan)

The enforced and regularised nature of these kinds of interactions meant that people's experiences as a ‘failure’ were frequently reiterated and embodied in their everyday lives, significantly undermining self‐worth and exacerbating mental distress. Yet, as the following quotes demonstrate, even attempts to ‘improve’ themselves by keeping well, looking after their appearance, or giving themselves an occasional ‘treat’ were felt to be a brash over‐indulgence that would be harshly judged by others, and undermine any claim to be considered worthy recipients of support.

I'm really, really struggling with my depression. I want to go away – but even if I find any money to go away, even for a weekend, if I just stay in a hotel by the sea, somewhere cheap, not even in season, I'm going to be judged aren't I, because I've spent that money that way. Even if I can claw that money together, they won't see it as a fact that I'm trying to get myself mentally well. (Siobhan)

We don't smoke, and we don't drink and we can't afford to go out – yet people will frown at us for the fact we've had a take‐away, It's like, well what can we spend our money on? We're not sat out there having a cigarette, we rarely drink, we don't do recreational drugs […] and yeah, we order a cheap take away, damn us, we're evil  (Myra)

Discourse around poverty‐related shame is often gendered and emphasises ‘embodied deficiencies’ (often relating to dress, weight and complexion) that are predicated on implied behavioural faults such as excess drinking and poor diet (Valentine and Harris 2014). Such discourse also relates to how money is spent, with attempts by people to avoid shame, be considered respectable, or protect their children by buying particular accessories and clothing often mocked or openly disapproved of (Jones 2012, Peacock *et al*. 2014). Cohering with other literature (e.g. Skeggs 1997), women in particular, felt that they were judged harshly, as Jessie's felt the need to moderate her actions makes clear.

If I go to the Job Centre, I won't take that handbag, that Ted Baker – it's only, my friend bought me for my birthday, I think they're 20 quid. Sometimes, walking around with that, I think if people know I'm on benefits they think ‘oh why's she got a bag like that? (Jessie)

### The normalisation of poverty‐related distress

Despite the clearly negative impacts of these narratives, we found abundant evidence of people embodying the mental distress of the narratives of blame and failure to which they were exposed. For Stella and Toni for example, turning to the welfare system at a time of crisis was felt to be a sign of personal weakness and failure:

I spent a huge amount of my time bed‐ridden and I think a lot of depression then, it wasn't the fact that I was bed‐ridden, it was the fact that I wasn't making these targets … the pressure that I'd somehow … failed … and the reason I was ill, I couldn't even turn my head let alone wipe my own arse, it was irrelevant to the situation – I just wasn't good enough. (Toni)

Those feelings of guilt were really strong – like ‘I can't believe I've given in’. ‘I can't believe I've done this’, and ‘[I've] just gone and done what everybody else does’ and ‘oh it's too difficult’ and I've thrown in the towel. It was that sort of self‐hatred almost of – you've become one of them – one of those people who do that, sit on the dole all their life sort of people’. (Stella)

In consenting to dominant narratives around their failures, participants commonly dissociated themselves from those seen as ‘scroungers’ or as ‘undeserving’ and subscribed to dominant rhetoric that both denied the existence of real poverty, whilst also morally condemning ‘the poor’ – as a result, perpetuating symbolic violence through enabling and propagating the very narratives by which they (the poor) were subjugated.

A further outcome of this complicity was the reluctance some felt to involve themselves in the welfare system, even when they were entitled to receive support. In such instances, people were keen to avoid welfare or to use services such as food banks precisely because they did not want to be perceived by others as the ‘type of person’ who would have to resort to this form of support:

I've been on ESA for 8, 9 years and I've never claimed DLA or PIP because I feel enough of a scrounger now the way the media has portrayed us […] every time you go and sit in front of somebody you're being judged. (Pam)

I don't want to be seen as a scrounger, but then again, I've paid into the system for years because I've always worked and worked properly and paid tax and all. But I've got this mental block about claiming any more. It's stupid, but … and no matter how many times I say to myself it's stupid, I still can't do it. (Kim)

Again, whilst this kind of denial can be seen as a form of self‐preservation in which people attempt to dissociate themselves from the stigma of poverty, such acts also make people complicit in broader narratives that deny the existence of ‘real’ and widespread poverty, and paradoxically, undermine the need for such support mechanisms to be endorsed whilst fuelling narratives that exacerbate mental distress.

The complexities of engaging with the welfare system also acted as a form of violence that adversely affected people's mental health, particularly when the ‘system’ endorsed a ‘guilty until proven innocent’ approach to assessing claimants. The need to attend regular appointments (which do not take into account unreliable and costly public transport, caring responsibilities or medical appointments), to provide sufficient evidence of job seeking, to engage with unfamiliar and often inaccessible terminology (requiring access to computer and Internet in line with the government's ‘digital by default’ system) for limited and usually precarious work opportunities, to keep in line with the array of changes to benefits and associated rules that have been enforced in recent years, to deal with and challenge (often using lengthy, complex and jargon‐laden forms) what many described as frequent under‐ or delayed payments or benefits sanctions, to be shown to be ‘bettering’ yourself through voluntary work placements and to be expected to display the necessary ‘work ready’ psychology (see Friedli and Stearn 2015) to convince benefits advisors of your credibility were just some of the challenges that people felt added to their mental distress, and ultimately resulted in them denying themselves their legal entitlements.

I avoid benefits like the plague, cos it's paperwork, getting asked stupid questions … the fact that you've got to go through all the paperwork and the tribunal and its like ‘you must be telling lies, we'll start off at that point. (Jamie)

I'm not on Jobseeker's [allowance] – that was a choice because I don't want to be called in and [they] say ‘are you looking for a job?’ – when I know I'm not mentally capable of looking for a job or physically capable. I am currently in the process of being diagnosed with fibromyalgia, but again, I'm on the process line for that, it's going to take some time. I go into the jobcentre […] basic questions sort of stuff they want to fish out – ‘have you looked for jobs? Have you got your proof? And sometimes, I'm not capable of looking or wanting the stress of trying to look for a job with two young children. (Chloe)

The requirements and expectations of other core service providers also acted as a key source of stigma and shame. Many families with young children, for example, were in contact, at some level, with various agencies such as housing providers, social workers and Children's Centres, who, according to their official remit, and to those who referred them to these services, were there to provide support to them. Yet, despite the often positive intentions of the service provider, the context of risk management and surveillance that now dominates the provision of much social support in the UK (Gupta and Blumhardt 2016), meant that these interactions frequently became dominated by discussions that questioned the lifestyles and actions of parents rather than identifying how best the service could help them. Parents interviewed explained how these encounters frequently led to them to question their parenting capabilities, and explained the impact this had on their mental health.

When you look around and you think everybody else is doing it, everybody else can cope with dealing with a child, why can't you, and it's just one of those things where you beat yourself up and you think if everybody else can do it why can't you, what is wrong with you and you just get worse and worse. (Sally)

That's where my depression comes out. Am I good enough [for my children], am I impacting them, am I screwing up? (Justine)

Here therefore, the dominant criterion of evaluation that parents could use to understand their lived experience of poverty (i.e. that of a risk to their family), was one that disempowered them even further by making them question their ability to be good parents rather than one that acknowledged the difficulties of raising children in poor quality, often overcrowded housing, with little available financial capital to purchase good quality food, or to engage in social and leisure activities.

### Medicalising poverty‐related distress

As the previous sections have discussed, everyday experiences of symbolic violence exacerbated feelings of shame, and worthlessness, with profoundly negative impacts on people's mental wellbeing. This in turn, influenced the ways that participants embodied their distress and sought to respond to it. Study participants described how dominant narratives of self‐responsibility placed pressure on them to legitimise their distress to family, friends and service providers, and that this influenced the way that they made sense of their distress as a pathological problem requiring medical treatment rather than an issue that primarily stemmed from broader economic and social inequalities. In such situations, people explained how they were actively encouraged to seek medical help by friends and family, or had themselves invested in narratives around the ‘normalcy’ and ‘need’ to see and act upon themselves as if they were ill and in need of treatment. This played out further in the consultation with the GP, and the acceptance of medication. For Sylvia for example, accepting medical support for mental distress due to issues relating to financial pressures and social isolation had been pushed as ‘the right’ and ‘responsible’ thing to do, with a refusal to do this not only likely to be judged as irresponsible, but also to result in lack of sympathy and potential exclusion if she was seen to be turning down her chance to access widely available treatments. Reflecting on this, she explained,

I felt that if I turned around and said ‘well I don't want the tablets, then they would probably turn around and go ‘well you're not that depressed then are you […] ‘I took them [antidepressants] for 3 months, just to keep, to pacify people really […] It was very much, I think if I'd been prescribed the pills and I hadn't taken them it was like, ‘well, you were offered help and you didn't take the help’.

Similarly, for Anna, taking antidepressants had felt like the right thing to do in order to make herself a better mother and partner, and to minimise risk to her child.

I'll take these because the doctor's prescribed them for me, I must need them, it must make me a better mum and it must make me this that and the other. And it didn't make me a better mum or a better girlfriend but at the time I thought it did, because the doctor prescribed them so he must think I need them […] I felt like I was doing everything I should be doing and everything I was told I should be doing and it felt very like, like you were told you had to take them because otherwise your baby won't be safe.

Alongside these pressures, the very fact that qualifying for key forms of welfare increasingly requires people to evidence some form of ‘legitimate’ health concern raises important ethical questions over the ways in which people are encouraged to accept a more passive, ill and ‘defective’ role in order to justify and obtain support. The implications of this kind of narrative can be seen clearly in Hansen *et al*.'s (2014) research in the United States in which the dismantling of traditional welfare payments and the establishment of a system that is increasingly medicalised and pathologised has pushed people to seek a clinical diagnosis and to accept potentially harmful medications in order to qualify for welfare support, whilst at the same time, fostering a legitimised version of ‘responsibility’ and respect amongst those struggling to get by.

In our research, it was evident that people frequently engaged with medicalising and pathologising terminology to explain and legitimise their experiences of distress. This kind of language was then reinforced and normalised through interactions with GPs and usually led to the prescription of antidepressant medications or talking therapies. Whilst this situation was facilitated through the authority and symbolic capital held by GPs (McDonald 2014), those we interviewed explained how they felt frustrated that they had become caught up in politically driven agendas around welfare entitlements, and most reported a felt need to try to protect their patients from the fallout from this. This could be through helping patients complete welfare administration forms, but more commonly manifested through the proffering of mental health diagnoses and treatment to enable the patient to legitimise their welfare claims, even in cases when the GP did not see the issue as inherently medical.

I will say this [depression and anxiety] is what I'm going to put on the form, but I know in my heart of hearts that it's not a medical problem. (GP)

Whilst GPs clearly had good intentions to support their patients, a number of factors worked together to ensure that existing inequalities were reproduced and became normalised within the consultation room. First, GPs in the UK are trained to recognise behavioural factors in health as they relate to issues such as lifestyle and diet. GPs are also generally trained to diagnose between *either* physical or mental health issues rather than recognising the interconnections between physical, mental and social factors. Thus without being trained to see the unequal economic structures and societal prejudices that shape the suffering of their patients, some health professionals tend to see mental distress as caused by either pathological issues related to brain ‘dysfunction’, or as caused by the patient's behaviour and ‘lifestyle’. This in turn leads to responses that promote what are often ineffective and potentially harmful medical treatments in the form of antidepressant drugs, or that blame patient distress on feckless and irresponsible behaviour without acknowledging the inequalities that cause this distress in the first place. As Holmes (2007, 2012) has argued in his work on migrant health in the US, such actions then inadvertently depoliticise suffering and in turn, reinforce and naturalise the very structures that uphold and reproduce oppression, resulting in the core determinants of distress going unacknowledged and unaddressed.

Even when GPs in our study did recognise the social and structural determinants of distress, the pressures faced to help a patient within the short timeslot of a consultation and the sense that there was little else they could do to alleviate suffering in what they saw as a hopeless situation tended to lead to a medicalised response,

I think the perception [amongst GPs] is that something like fluoxetine is a very safe, fairly clean, drug, and won't do very much harm. So it feels like a kind thing to do if you've got somebody in a situation that can't be changed – to prescribe them with medication that makes them feel slightly better about their situation. There isn't good evidence to support it, but we still do it. (GP)

You know by doing that [prescribing antidepressants] you feel that at least you tried to give something when you can't change their … you can't give them a roof over their heads, you can't change the fact that they don't have any support or family around. (GP)

There was also evidence to suggest that GP responses to patients experiencing poverty‐related mental distress were mediated through perceived differences in the capabilities of their patients, again serving to reinforce negative stereotypes and narratives. Despite their small effect size for what is clinically classed as low‐level depression and anxiety, some GPs explained how people experiencing poverty‐related distress would ‘cope’ better with antidepressants than talking therapies, the latter requiring a level of emotional labour and commitment that they did not feel reassured that the patient would be capable of giving. In reference to low‐income patients, one GP explained:

It's jolly easy to go and see your GP and get a prescription of some kind of pill and it's much harder to actually go to [therapy provider].[…]. I think it's a much easier option to go and see the GP and get medications.

Any harmful side effects from the medications were therefore moderated and justified by a perceived lack of ability amongst patients to commit properly to alternative options. Once prescribed with these medications, it was common for people to remain on them for years, sometimes decades, with what they felt was little opportunity for review or support to stop.

### Resisting dominant narratives

As the previous discussion has described, the everyday forms of symbolic violence inherent in narratives of responsibility were found to exacerbate shame and stigma and in turn, influence the medicalisation of poverty‐related mental distress. Although Bourdieu has been criticised for his harsh depiction of working class cultural dispossession and passivity (see Skeggs 2004), *The Weight of the World* includes case studies of individuals who are consciously alert to processes of discrimination and who exercise agency in engaging in acts of resistance against this.

Examples of such agency were also evident within our research when participants actively took on the notion of responsibility, but repurposed it in a way that they felt distanced them from external judgement and gave them more control over their identity and actions. This frequently involved people surrounding themselves with a tight‐knit social support network with whom they could share their concerns and experiences, and in so doing, help boost their mental wellbeing. A key characteristic of these self‐organising and informal support groups was their refusal to operate as ‘official’ third‐sector groups – in some cases, deliberately not seeking external funding so as to avoid bureaucracy and the requirement to ‘tick boxes’ which they felt spoke to someone else's (middle class and usually deficits‐oriented) agenda.

Yet, although some level of satisfaction was evident in the ways that people resisted or repurposed the dominant narratives and social hierarchies to which they were exposed, such acts ultimately did little to change their broader subjection to forms of symbolic violence and domination. As Bourdieu and Wacquant (1992: 82) explain, ‘the dominated, in any social universe, can always exert a certain force, inasmuch as belonging to a field means by definition that one is capable of producing effects in it’. However, because active resistance colludes with the reproduction of existing hierarchies, ‘the dominated seldom escape the antinomy of domination’. Explaining this through a case study of resistance to the school system, Bourdieu and Wacquant (1992: 82) emphasise how choices to resist or acquiesce are both equally problematic and are likely to end up entrenching marginality:

To oppose the school system, in the manner of the British working‐class ‘lads’ analysed by Willis (1977), through horseplay, truancy, and delinquency, is to exclude oneself from the school, and increasingly, to lock oneself into one's condition of … [domination]. On the contrary, to accept assimilation by adopting school culture amounts to being coopted by the institution. The dominated are very often condemned to such dilemmas, to choices between two solutions which, each from a certain standpoint, are equally bad ones.

In our research, many parents had, over time, responded to the frameworks of risk in which they were judged by entirely removing themselves from the systems that were, in theory, meant to support them. Parents we spoke with explained that this enabled them to ‘take responsibility’ in a way that they felt gave them more control, resist the ongoing judgement and moralising they felt they were subjected to, and feel ‘safer’ in terms of preventing opportunities for their children to be removed into statutory care. However, refusing support out of fear can also be seen as an outcome of symbolic violence, in which people's attempts to counter the status quo ultimately results in their exposure to further forms of suffering – both in the ways that they are categorised by state agencies as (even) more of a risk to their children, with associated and potentially severe consequences; and in denying themselves the means by which they and their families can benefit from the support to which they are entitled.

We also encountered cases where people had resisted the medicalisation of poverty‐related distress through refusing or discontinuing antidepressants or talking therapies. Again though, a refusal to subscribe to the mental health treatments on offer fuelled stereotypes relating to dominant notions of ‘responsible’ behaviour (as in Sylvia's quote, above), and in some cases, a breakdown of trust with their GPs, resulting in people withdrawing from the very health system that was meant to assist them.

## Conclusions

Narratives of self‐responsibility have been amplified during the current period of austerity and welfare reform in England. Whilst not in themselves unique, their positioning alongside changing conceptualisations and treatment strategies for common mental health issues has a range of very real and adverse implications for some of the nation's poorest communities. The concept of symbolic violence enables a useful lens to understand how such narratives can become embedded and embodied in ways that serve to uphold, normalise and reproduce dominant power hierarchies, health inequities and poverty‐related mental distress – through aggravating feelings of worthlessness and shame, morally condemning the ‘poor’, and deterring people from claiming the welfare support to which they are entitled.

At the same time, we have shown how, when under pressure to legitimise their distress, people invest in biomedical narratives that question their sanity and their ability to cope, enabling their experiences of poverty‐related distress to become recast as pathological issues requiring medical solutions. This situation was further normalised when GPs, already under extreme time and resource pressures, put forward medical or behavioural explanations that inadvertently blamed the patient for their distress, or when they prescribed antidepressants as an act of ‘kindness’ to help numb the painful everyday realities of poverty.

Whilst we encountered examples in which dominant narratives of responsibility were resisted or repurposed, and which gave people some sense of control and solidarity, such acts ultimately did little to change their broader subjection to forms of symbolic violence and domination. Indeed, such cases frequently resulted in people withdrawing or alienating themselves from the very systems that were in theory meant to support them.

At the heart of the issues raised in this paper are questions around where responsibility for mental health and wellbeing should lie, and how progressive and emancipatory change might be realised. Bourdieu's work is inherently pessimistic in this regard, raising challenging questions around the relationship between his political activism and his academic sociology. Whilst dominant discourses often silence more marginalised voices and perpetuate psychological suffering, it is systemic and structural changes that are needed to ensure that these voices are actually heard and accorded agency. This means a move away from vertical, top‐down forms of power to forms of organisation that are horizontal and relational (see Cottam 2018) and that create and engage with new cultural narratives to foster positive transitional change (Hinchliffe *et al*. 2018, Thomas and Fietje 2020).

Governments can facilitate responsibility in citizens when they provide the material and structural resources required for this to become feasible and when they do so in a way that is respectful and emphasises people's self‐worth. Yet, within the current neoliberally oriented era, government and popular rhetoric around individual responsibility continues to act as vitriolic narratives that exacerbate blame and mental distress and deflect attention from the responsibilities of those with the power and remit to effect positive change.

## Data Availability

The data that support the findings of this study are available to users registered with the UK Data Service, reference 10.5255/UKDA‐SN‐853788.
